# It is a relay not a sprint! Evolving co-design in a digital and virtual environment: neighbourhood services for elders

**DOI:** 10.1007/s43508-022-00053-y

**Published:** 2022-11-03

**Authors:** Stephen Osborne, Madeline Powell, Maria Cucciniello, Joanne Macfarlane

**Affiliations:** 1 Chair of International Public Management and Director of the Centre for Service Excellence (CenSE), University of Edinburgh Business School, University of Edinburgh, 29 Buccleuch Place, Edinburgh, EH13 9PH Scotland, UK; 2 Marketing, University of York Management School, Heslington, York YO10 5DD England, UK; 3 Public Management, Bocconi University, Via Roberto Sarfatti, 25, 20100 Milan, MI Italy; 4 Research Fellow, Centre for Service Excellence (CenSE), University of Edinburgh Business School, University of Edinburgh, 29 Buccleuch Place, Edinburgh, EH13 9PH Scotland, UK

**Keywords:** Public services, Vulnerable adults, Co-design, Virtual co-design, Public service logic, Elderly services

## Abstract

There is an emerging body of research on the co-design of public services, including co-design with vulnerable adults. However, what has been less explored has been the impact of digital technology and virtual environments upon the co-design process in this context. This paper analyses the contingencies of virtual co-design through a case study of a project to develop supportive local communities for vulnerable elderly people. This project was initially planned to use traditional co-design methods within a face-to-face environment, in the context of the local public service ecosystem. The CoVid-19 pandemic made this impossible. Consequently, an innovative approach to co-design was developed that shifted the process from a face-to-face to a virtual environment. This exploratory paper reports and evaluates this approach and its implications for the future of the theory and practice of the co-design of public services for vulnerable adults. *Theoretically* the paper evolves a model of co-design in a virtual space that is embedded within a public service ecosystem framework of value creation. *At a practice level,* the paper provides insight into the strategic and operational management of co-creation in a virtual space. It evolves the ‘Relay’ model of asynchronous co-creation across time and considers it key contingencies.

## Introduction

This paper analyses the co-design of local neighbourhood services for vulnerable elderly people. It is based upon the design stage of a major project in Scotland to develop a supportive community approach to the needs of vulnerable elderly people. The project commenced in early 2020 and was planned to utilise ‘traditional’ face-to-face (F2F) co-design approaches for public services. These approaches have their own challenges for working with vulnerable adults, such as the impact of cognitive impairment and the resolution of differing perceptions of need across different stakeholder groups—including the elderly person themselves and their friends and/or family (Mulvale et al. 2019). Consequently, the initial co-design approach had three features. First, it took a public service ecosystem approach to local neighbourhood services (Osborne et al., 2022; Petrescu, 2019) that situated the needs of vulnerable elderly people within their institutional, service and individual contexts. Second, it explored the interface between the phenomenological experience of a community service by an elderly person and its impact upon their needs and expectations. Third, it utilised existing experience on the co-design of public services, particularly with vulnerable adults (e.g. Scarli, 2021).

The onset of the CoVid-19 pandemic profoundly changed the co-design process. Most strikingly, it shifted the process from a F2F experience to a virtual one, prompting the potential for innovative developments in theory and practice. An initial scoping exercise confirmed that little literature on virtual co-design existed to guide this migration (Kennedy et al., 2021). Consequently, this process was challenging not only for the co-design team but also for the service users involved. This paper explores the model of virtual co-design that was evolved in this context and considers its implications for the theory and practice.

The first part of the paper will explore the existing literature across four dimensions:

- The nature of PSEs and their implications for co-design,
- The co-design of public services,
- Co-design in a virtual environment, and
- Co-design with vulnerable adults.

The second part will report the evolution of our approach to co-design and its migration from a F2F to a virtual environment. The final part will evaluate this approach and consider its implications for theory and practice.

## Literature review

### The public service ecosystem (PSE)

The concept of the ‘service ecosystem’ initially developed in the work of Steven Vargo and his colleagues on the ‘service-dominant logic’ approach to service management and marketing (SMM). It drew upon the analogy to ecosystems within the ecological literature that has developed from the work of Tansley. Service ecosystems have subsequently become the front-line of SMM theory development, integrating institutional and user concerns with the service level (e.g. Mustak and Ple 2020, Vink et al., 2021).

Systemic approaches to public service delivery, such as the ‘Production of Welfare’ model of social care (Knapp, 1984) have also long been a pre-occupation of the public administration and management (PAM) research community, as has context (Pollitt, 2013). The PSE approach goes further, however. It explores both context and system and the interactions of the institutional, service and individual levels of public service delivery. Trischler and Charles (2019), for example, have described it precisely as a unifying framework through which to understand the complexities of public service delivery and value creation at the societal, service, and individual levels. Value is not something created in isolation by public service users. Rather it is nested within overlapping and interacting relationships within the PSE.

This argument was amplified further by Petrescu (2019), and Strokosch and Osborne (2020) also offered an empirical exploration of PSEs in a UK context. They concluded that, within PSEs, value is shaped by the interplay between all of the dimensions of the ecosystem, and not least by the wider societal context and the values that underpin it.

Rossi and Tuurnas (2021) have subsequently argued that PSEs reveal the complexity of value creation conflicts, whilst Kinder et al., (2020, 2021) have argued that PSEs have now replaced networks as the most persuasive framework for understanding public service delivery. Studies of PSEs can now be found exploring such diverse issues as digital public services and Big Data (Cordella and Peletti 2019), smart cities (Ciasullo et al., 2020), value destruction within public services (Engen et al., 2021, Tie and Osborne, 2022), and public service responses to the CoVid-19 pandemic (Brodie et al., 2021). It has also been integrated into formal PAM theory through the development of Public Service Logic (Osborne, 2021) with a focus on the elements of value in a public service context (Osborne et al., 2021).

This current paper argues that PSEs provide an essential context within which to understand public service co-design and which will allow us to integrate and advance both PAM and co-design theory and practice. It adopts and adapts the frameworks of Trischler and Westman-Trischler (2021) and Osborne et al (2022), as discussed below. Rather than designing discrete services, the project here sought to design a supportive community ecosystem for vulnerable elderly people.

### Co-designing public services

#### Service design and co-design

It must be emphasised that an immersive exploration of service design and co-design and its application to public services is beyond the scope of this paper and exists elsewhere (Bason, 2017). Here, we examine briefly the basics of service design and its evolution before exploring its application to public services.

The discipline of service design evolved from the work of Shostack (1982). Increasingly, service design theory has explored the links both between service delivery and value creation for service users (Yu & Sangiorgi, 2018) and between service design and innovation (Patricio et al., 2018). Moreover the focus has shifted from service design *by* experts and towards co-design *with* service users (Trischler et al., 2018). Embedded within this is a focus upon value creation as the core element of service delivery. Whilst early service management theory had focussed upon service production and co-production, the focus latterly has moved to the use and consumption of services and the means through which ‘service’ can add value to the lives of service users (Gronroos & Voima, 2013; Vargo et al., 2008). Establishing the links between service design/co-design and value creation has thus become at the forefront of the service design field (Andreassen et al., 2016).

#### Public service design/co-design within the PSE

Early examples of public service co-design did not draw explicitly on the service design discipline (Farrell & Goodman, 2013). By contrast, the Scottish Government has drawn explicitly on service design theories to develop an ethnographic approach. They adapted in particular the work of the Design Council on the ‘double-diamond’ approach to service design (Council, 2007). They argued that

‘The design process reminds us that we have to be sure we’re creating the right thing before we can design something that’s fit for purpose and meets the needs of users, staff or organisations’ (Scottish Government, 2019, p. 14)

Subsequently, the last decade has seen a growth of the application of explicit co-design principles to public services, and increasingly predicated on the principle of co-design for value creation (*inter alia*, McColl-Kennedy et al., 2012, Donetto et al., 2015, Bradwell & Marr, 2017, Blomkamp, 2017, Dudau et al., 2019). Key issues in this evolution have included the implications of power imbalances for co-design (Farr, 2018) and the links between co-design and social innovation (Voorberg et al., 2015; Whicher & Trick, 2019). The most recent developments in public service co-design have positioned it within the PSE. Osborne et al. (2022) conceptualise PSEs across four levels:

- *The macro-level of the institutional context of public services* (societal beliefs, norms and rules)—often codified within public policy,
- *The meso-level of the service context of public services*, including the local community, networks of public service organisations, and the processes of public service delivery themselves,
- *The micro-level of the individual context of value creation*, including public service users and their other key stakeholders (such as friends, families and carers), public service delivery staff, and citizens (for example, as volunteers), and
- *The sub-micro of the individual and professional beliefs of the key stakeholders*, above.

This project sought to work across all four dimensions—by challenging societal beliefs about the capacity of elderly people, by creating an integrated network of service that focussed on value creation within participants’ lives, by exploring what value the key stakeholders were seeking to create, and by questioning individual and professional beliefs about the capacity of elderly people.

### Co-designing public services in a virtual environment

The Covid-19 pandemic has amplified citizens’ use of the Internet and has led to an unparalleled surge in the use of digital technologies across society. Public services were required to move online due to the social distancing measures imposed by governments. This digital surge has impacted the co-design of public services as it has other elements of such services. Co-design has historically emphasised F2F engagement, but the pandemic required the translation of co-design from a F2F to a virtual process. To date, few studies have investigated such public service co-design in a digital and virtual environment (Dietrich et al., 2018)—though some early studies are emerging (e.g. Dietrich et al., 2021; Kennedy et al., 2021; Martinez et al., 2021; Micsinski et al., 2021). This present paper is a contribution to this emergent literature.

A few lessons do exit from what prior studies have been carried out. In relation to design tools and methods, for example, Shi et al (2015) evaluated an early digital co-design approach to hospital design. They argued that public service users can find traditional approaches to co-design too abstract and difficult to engage with, whilst a virtual approach was found be more accessible—a view shared by some private sector researchers (e.g. Nambisan, 2002). Apostolou et al. (2011) have argued that virtual co-design can offer participatory virtual platforms for public service stakeholders to collaborate. However, they have also underlined the need for this to be ‘customised’ within the specific PSE of distinct user groups rather than as a ‘one size fits all’ process. Finally, Kennedy et al (2021) also argue that virtual co-design for public services can be highly cost effective—though this point remains debated.

### Co-design with vulnerable adults

The impact of involving service users in the co-design of public services is being increasingly recognised, as is its potential to empower service users by placing them at the forefront of the design of their services (Scarli, 2021, Mulvave et al. 2016, 2021). However, enabling such co-design can be challenging (Mulvave & Robert, 2021). The overarching term “vulnerable”, for example, is contested by both users and professionals, who have argued that the distinctive needs of discrete user groups must be recognised (Katz et al., 2020). Developing approaches to engage vulnerable adults in co-design can hence be challenging, as tools and methods that might work for adults with mental health conditions might not be as effective on adults that suffer from cognitive impairments (Hendriks et al., 2015). Moreover, tools which are used in many ‘standard’ co-design toolkits can be inappropriate for vulnerable adults, who can struggle with both sensory skills (verbal, written and visual) and higher cognitive ability (such as expressing abstract concepts) (Bourazeri & Stumpf, 2018; Rodgers, 2018).

Four principles emerged from the literature on co-design with vulnerable adults that became core to this project. First, there are challenges in accessing and engaging such individuals, particularly in relation to overcoming stigmatisation. Research has shown that vulnerable adults, whatever their needs, are underrepresented in the co-design process (Moll et al., 2020). Further, vulnerable adults who have been marginalised or subjected to stigma throughout their life often avoid participating in the co-design process due to the fear of being further stigmatised and/or their opinions not valued. There are also issues of trust as co-design professionals can be seen as ‘outsiders’, thus making access challenging (Amann & Sleigh, 2021). Consequently, vulnerable adults can require support in building their confidence to participate in co-design and to feel a sense of ownership over the co-design process (Bourazeri & Stumpf, 2018).

Second, there are issues in the use of support staff to facilitate co-design with vulnerable adults. Such support is essential but can also become a block in its own right if the participants experience it as replacing or re-interpreting their views and experiences (Brereton et al., 2015; Hendriks et al., 2015). Thus, care had to be taken in this project to ensure that participants’ viewpoints are being represented rather than replaced.

Third, the significance of power imbalances has to be addressed in co-design with vulnerable adults. These can significantly damage open communication and a willingness to participate (Moll et al., 2020). Successful co-design can challenge and shift these existing power imbalances and the marginalisation of vulnerable adults. Unsuccessful co-design can exacerbate them (Mulvale et al., 2021).

Fourth, there is also an emerging literature on co-design of public services with elderly people in particular. This literature emphasises.

- the significance of sensitising both the co-design process to the context of elderly people and elderly people to the processes of co-design (Aidemark et al., 2020),
- the need to evolve co-design approaches that do not rely upon participants possessing sophisticated cognitive skills (Botero & Hyysalo, 2013),
- the importance on focusing on the processes rather than the artefacts of community care services (Island & Lundh, 2018), and
- the impact of power imbalances between elderly participants and care staff in co-design processes (Wright et al., 2017).

This literature also confirmed the significance of situating co-design within the PSE of vulnerable elderly people rather than seeking to apply a generic model and process of co-design (Camarinha-Matos et al., 2015). This was a core principle for this project.

### Interim conclusions for the literature review

This literature review confirms the potency of co-design to developing effective services for vulnerable elders, and to situating this within the PSE of these adults. Equally it highlights potential challenges of co-design with vulnerable adults in general, and elderly people in particular. It has also highlighted the potential and constraints of a virtual approach to co-design for vulnerable elderly people. The rejection of the use of new digital technologies and social media can be especially prevalent for elderly people with low income and/or poor education (Papa et al., 2017). The remainder of the paper will explore and evaluate the evolution of our model in responding to these challenges.

## The Peoplehood project

### Policy context

The *Peoplehood* project aspired to develop sustainable and supportive local neighbourhoods for vulnerable elderly people. It was led by Blackwood Housing and Care (‘Blackwood’) and was part of the United Kingdom (UK) government’s *Healthy Ageing Programme* to promote innovative approaches to services for elderly people.^1^ This programme was established to encourage and support elderly people ‘to remain active, productive, independent, and socially connected across generations for as long as possible’.

At the macro-level, this programme addressed three core societal themes in the UK. These were the level of unmet need for public services for elderly people (Walker, 2018; Westwood & Daly, 2016), the escalating cost to government of traditional forms of support for elderly people (National Audit Office, 2016), and the need to challenge prevailing societal attitudes towards elderly people and their contribution to society including the import of the ‘silver economy’ (Abrams et al., 2015, European Commission, 2018).

*Peoplehood* took an explicit PSE approach that sought to create change at the macro, meso, micro, and sub-micro levels of the elderly care ecosystem. It was also predicated on ‘value creation’ within this ecosystem rather than ‘service delivery’ imperatives. This was informed significantly by the Public Service Logic model of value creation in public services rather than internal value chains and a preoccupation with internal organisational efficiency (Osborne, 2021).

### Local context

It is important to emphasise that the Peoplehood project was located in Scotland, which has developed a unique response within the UK to public services reform, since UK political devolution (Wallace, 2019). Specifically, the recommendations of the influential Christie Report on reforming public services (Commission on the Future Delivery of Public Services, 2011) have emphasised the centrality of co-design and co-production to public services in Scotland.

*Peoplehood* was located in three distinctive rural/urban/suburban regions in Scotland and included partners in the for-profit, public, and third sectors. It aspired to develop supportive neighbourhoods for elderly people that addressed the five key issues identified in the Healthy Ageing agenda: designing age-friendly homes, sustaining physical activity, managing common complaints of ageing, supporting social connections, and creating healthy and active places.

This present paper reports and evaluates Stage I of the project that worked at the sub-micro and micro-levels with elderly people and their key stakeholders to co-design a model of support for them. Stage II of the project began in late 2021 with the focus shifting to the meso- and macro-levels. It will work over three years to challenge prevailing attitudes about the capabilities of elderly people and to create supportive local ecosystems in each of the localities, with the project partners working in collaboration with elderly people to enact the co-designed service model.

### Evolution of the co-design project from F2F to virtual co-design

The project was approved and funded by the UK Government in early 2020. It anticipated utilising existing F2F methods of co-design and drew upon the Design Council’s (2007) ‘Double Diamond’ approach to co-design. The co-design was led by the Centre for Service Excellence (CenSE) at the University of Edinburgh, of which the authors of this paper are all members, together with Blackwood staff. Iterative workshops were planned in each locality where participants (both elderly people and support/care staff) would work together in small groups to explore their needs across the five themes identified above. These insights would subsequently be integrated into a project proposal for implementation as part of Stage II of *Peoplehood.*

The reality of the project was very different from this, however. One month after project approval, the CoVid-19 pandemic became a global reality. The project team, therefore, had to migrate the entire co-design process to a digital/virtual environment and with no F2F contact either within the project team or with/between the elderly participants. This was undiscovered territory for co-design within a public service context.

Five principles, developed from the above literature, guided this virtual re-orientation of the co-design process. First, perforce it would involve less individuals than had been the original intention, because of the limitations of the extant virtual technology. This limited the number of participants who could be comfortably involved in the virtual co-design workshops whilst still feeling engaged with the process. Second, and because of the above, the project sought to identify a smaller group of ‘lead users’, who had extensive experience of the health and social issues that the overall programme was based on. Third, the project was a microcosm of the pre-dominant experience of elderly people, in that the level of their digital skills was lower than for the broader population. Consequently, this implied significant support needs to enact the co-design process successfully in a virtual environment. Fourth, the process needed to be iterative, to explore emergent issues both within each neighbourhood and across the thematic strands, as well as being integrated into a whole at the project level. Fifth, because of the prior experience of the constraints of the virtual workshop format, and the consequent marginalisation of elderly people within such workshops, the model created an iterative and serial sequence of workshops across the ecosystem of the project. Hence, the project took an explicit PSE approach to value creation for the participants. It sought to explore the interplay of societal, local community, service-driven, and individual perspectives on the potential value that could be created across the PSE by the project.

The first workshops comprised elderly participants in each locality, exploring their own needs and beliefs (sub-micro and micro-level). Their work was then passed onto a second set of workshops with Blackwood care staff, to explore the impact of their professional beliefs and the service implications (sub-micro and meta-level). The third set of workshops then passed their work back to elderly participants but this time working across the programmatic themes rather than in localities (micro-level). Finally, the outcomes of this iterative sequence of workshops was passed on to the strategic planners of the project to integrate with the strategic themes and priorities of the overall *Peoplehood* and *Healthy Ageing* programmes (macro-level).

The iterative model provided a check to reaffirm regularly that the main issues and challenges identified by the key micro-level stakeholders were being addressed. This model became known as the ***Relay model***, where each group of stakeholders passed on their work to the next group to refine and/or evolve. This was developed initially to deal with the constraints of the virtual format. Serendipitously, however, it was also found to be highly effective in addressing the power imbalances identified above as endemic to co-design. It allowed all voices to be heard within the co-design processes, without one voice ‘crowding out’ or drowning the others. It presented its own challenges, as discussed below, but proved an effective model of co-design within the virtual environment.

## The five stage Relay model of co-design

The Relay model that evolved comprised five stages. *Stage I* was the ‘pre-process’. This included gathering and synthesising existing demographic and secondary information on need in each of the three localities. This was followed by a series of meetings between strategic managers in Blackwood and the co-design team to clarify the co-design objectives, the technology available, and how to facilitate and frame the process for participants. Simultaneously, Blackwood worked through its network of neighbourhood managers to identify the ‘lead users’ who could engage with the project and both to brief them on the aims and expectations of the project and to sensitise them to its virtual environment.

*Stage II* comprised three iterative workshops in each locality—nine workshops in all. The first workshop focussed on the first of the Peoplehood themes above, whilst the subsequent ones covered two themes each. These workshops were held online in virtual design spaces and facilitated by use of the ‘Mural’ online design platform.^2^ The workshops included six–eight lead users in each locality. They comprised not only local residents but also other key stakeholders, such as family members.

In the project bid, Blackwood had pre-defined some ‘oven-ready’ options for the project. However, on the advice of the co-design team, these were not presented in the initial workshops, so as not to make assumptions about needs or potential responses. Instead, open-ended discussions around each theme were held. These covered both the experiences of workshop participants and what they perceived their needs to be in relation to each of the five themes. The Relay model explicitly avoided exploring resolutions to emerging issues at this stage, to keep the discussion as open as possible.

At this stage, Blackwood staff outside of the co-design team were not actively involved in the process, for three reasons. First, the project was concerned not to ‘over-shadow’ or intimidate local participants by the presence of too many professional staff. This is an issue in general for co-design, where articulate and enthusiastic staff members can, albeit unintentionally, inhibit/re-frame the contribution of elderly participants. This is a special concern in a virtual design space though. Here, the elderly participants have significant issues in acclimatising to the digital environment that would be exacerbated by the presence of professional staff with more familiarity with its contingencies. Second, and similarly, the project team were concerned to ensure that the service delivery pre-conceptions of service staff did not crowd-out the experiences and expectations of less articulate service users. Third, staff and local residents were also kept separate so that existing services or resource constraints did not contaminate the articulation of their needs by local residents, and staff did not overshadow users in virtual environment. This approach became a core element of the Relay model.

Significant practical difficulties were encountered during this stage of the process. Co-design is by its nature a time-intensive process. It requires time for participants to become used to an unfamiliar environment and to be encouraged to contribute, for example. This was exacerbated in a digital and virtual environment. Four factors were noteworthy in this context.

First, the technology itself is still somewhat unstable. It was common for participants to loose online connection, for example, or for the digital tools to freeze. The project team quickly discovered that the time required for each workshop was considerably greater than if they had been carried out F2F. Second, it had been anticipated that participants would lack substantial digital skills in a virtual environment and that time would need to be spent in upskilling them in these. This was indeed the case, though with incrementally increasing familiarity, speed of use improved with each workshop. Participants, however, did require iterative advice on how to use the tools in the virtual space.

Third, the Relay model does require significant staff input to support the engagement of participants. Dedicated staff members worked with the elderly participants, often populating the Mural board to their explicit instructions, and constantly checking that they were being accurate in their representation of views. This was to counteract the impact of low levels of digital skills of the participants. One participant, for example, could see the Mural board and could comment ‘in vivo’ but was not able to actually interact directly with the virtual design space. This required a dedicated support worker to act as their scribe. Whilst labour intensive, this did support maximum involvement of participants in the virtual space.

Fourth, the virtual environment itself was an intimidating one for participants, and could also be an isolating experience for them, at least initially. This could lead to participants becoming taciturn and reticent to contribute. The use of icebreaker activities assisted in minimising this factor, but it was a recurrent theme throughout. In the latter stages, after feedback from the group, the co-design facilitators opted to utilise a gallery view of participants as often as possible, because participants said that seeing other people’s facial expressions made them feel more comfortable when they were speaking and minimised the isolating effect of the virtual interaction.

In *Stage III*, the co-design team brought together the outputs from all nine initial workshops. These were then explored further with Blackwood staff in a series of five sprint workshops—again conducted in a virtual space. These took the needs and ideas identified in Stage II and evaluated them from a service delivery perspective. Again, traditional models of public service co-design would aspire to bring together the needs and experiences of both service users and service staff contemporaneously. This was avoided here, for the reasons discussed above. It avoided service staff crowding out service users in the discussions and also prevented the professional staff from moving directly to ‘oven ready’ models of service delivery without proper exploration of the actual experiences and needs of elderly people.

In *Stage IV*, the outputs from the staff sprint workshops were taken back to users. At this stage, the participants from the three localities were combined into one group. This was primarily to gain a holistic view of needs across the three localities—and with some participants self-selecting the themes that were most relevant to their own experiences and needs. There was also some drop-off in attendance at the co-design sessions, reflecting the reality of digital fatigue in a virtual environment. Participants reported that they found the virtual sessions extremely tiring because of unfamiliarity with the virtual environment and the newness of the virtual co-design tools.

Stage IV thus required the participants to prioritise, on a holistic basis, the needs expressed in each community and also to consider the service proposals suggested by the Blackwood staff in their sprint sessions in Stage III. It prioritised the service offerings overall and illuminated not only some important distinctions between the staff and elderly resident perceptions but also between participants from the three localities. Blackwood also surveyed elderly communities across Scotland through its network at this stage, to test if the perceptions of the lead users did indeed chime with those of the wider community. This survey prompted over five hundred responses and demonstrated strong support for the ideas put forward by the lead users.

In *Stage V*, the Blackwood strategic service managers and the co-design team reviewed the co-design outputs and developed them into proposals for the *Healthy Ageing Programme’s* 2nd phase. These proposals have now been approved and funded and the focus has moved to implementation. A key element of implementation will be engagement with the key institutional stakeholders, such as the social care, health, and community/neighbourhood services. A more traditional co-design approach might have engaged with all three constituencies (service users, service staff, and institutional stakeholders) together. However, as has been emphasised, the reality of a virtual co-design space made such an approach highly challenging. The Relay model evolved here allowed all parties to contribute effectively to the process, and recognised the expertise of each group, but without one group crowding out the others.

## Evaluation of the Relay model of virtual co-design

Subsequent to the virtual design process, an evaluation of it was carried out by the CenSE team. This evaluation included online and in person interviews with the service users involved in the project, supplemented by a follow-up survey, interviews with the Blackwood staff^3^ who had been part of the process, and the key service managers responsible for overseeing the project. Key documents produced by project were also examined.

### Strengths

The virtual co-design process was challenging but brought real benefits to this project. Three were particularly important. First, the virtual environment minimised the impact of both location and disability upon involvement in the co-design process. It can be notoriously difficult to bring together participants from across diverse locations, whilst disabilities can prevent access to some co-design locations. The virtual environment meant that issues of distance or disability were unimportant—anyone with access to a computer was able to be involved in the project. This became a consistent theme across the project—of synchronous but spatially distanced co-design. It thus broke down physical barriers to co-design. Second, the digital engagement of elderly people was a key element of the UK *Healthy Ageing Programme*. By taking co-design into a digital and virtual environment, the project not only facilitated co-design but also tested out the challenges of digital engagement in real-time: the co-design process became a testbed for digital engagement as well as designing services to address it.

Third, the Relay model of co-design incorporated residents, family, and care staff—but involved them asynchronously. This approach emerged as a practical response to one of the constraints of co-design in a virtual environment—that engaging too many people in a virtual space can be highly alienating as the number of individual represented on a computer screen multiples. Further there was a clear imbalance between the digital skills of care and professional staff and of elderly participants. The elderly participants required significantly more time and support to engage in the project, compared to professional and care staff. Separating the two parties to the co-design process addressed these issues of power and skills. By enabling their own space, the virtual co-design process allowed the elderly participants to express their ideas more freely and not to be dominated by the pre-conceptions or enhanced digital skills of the care/professional staff. This issue of power asymmetry has been a consistent issue for co-design and co-production in social care (Farr, 2018). The Relay model evolved here addressed this imbalance. The inputs of the two parties were asynchronous in time, but built on each other iteratively. It allowed vulnerable elderly participants to be engaged in the co-design process but minimised the impact of extant power relationships between staff and service users.

### Challenges

Inevitably the migration of the co-design process to a virtual environment was not without difficulties. Five were identified in this project, primarily of an operational nature. First, the virtual format did limit the number of users who could be involved in a workshop. Significant resources and commitment were required to ensure that users could engage in the project, and this limited the numbers involved. The Relay model sought to minimise this effect both by focusing on lead users and by validating their work through a follow-up survey. Second, the digital context had its own challenges. Many users had poor digital skills and/or poor internet connections. This slowed the process and co-design and required substantial support of the users involved in the co-design process. The Relay model developed was a powerful co-design tool but not a cheap one—challenging the cost-effective expectations of some proponents of virtual co-design.

Third, because of the issues identified above, the co-design process took significantly longer than a F2F co-design process. This was because of the facilitation and support needs involved and because of the time required for the iterative and asynchronous phasing of the co-design process over time. As one participant noted humorously: ‘it’s a relay, not a sprint!’ This subsequently became the ‘tag-line’ for the project.

Fourth, as the process was worked through, an inevitable element of ‘Zoom fatigue’ set in. This led to a greater level of participant passivity and/or the loss of some of the users from the process. This was mitigated somewhat in the later stages of the project by the aggregation of the three geographic user groups into one larger group. Fifth, the migration to a virtual context for the co-design work did mean that the users involved were a self-selected group who felt at least moderately comfortable working in a virtual environment. This did have a skewing effect on user involvement. Again, the project sought to mitigate this impact by the inclusion of the subsequent wider validating survey.

## Conclusions: implications for theory and practice

Co-design has a substantive lineage in service management and has grown as a significant theme within the field of public services (Bason, 2017; Donetto et al., 2015). Dietrich et al. (2018) and Mulvale et al (2021) point to the potential for co-design to humanise public services and to empower vulnerable adult users of these services. Traditionally, such co-design has been carried out F2F in real-time with service users. This prevailing logic has been challenged profoundly by the impact of the pandemic. It has required the transformation of co-design from a F2F to a virtual process.

This paper has reported one example of this transformation in the context of designing supportive communities for vulnerable elderly people. Adapting a virtual co-design process with such vulnerable users was challenging but important. Users with more extreme needs (notably elderly people, the sickest or the most vulnerable) demand the most from public services, yet the very limited number of existing virtual co-design projects have focussed on users with ‘median’ needs, such as middle-aged people or people with ‘median’ health conditions (Amann & Sleigh, 2021). This project thus took virtual co-design into an undiscovered country.

It resulted in what we have termed the ***Relay model of virtual co-design***. This employed asynchronous engagement of elderly people and of care staff within the virtual design process. The model did have disadvantages—notably in the time taken over the process and in minimising learning from the interaction between service users and care staff. However, it had distinct advantages too. It minimised the impact of existing power differentials on the co-design process, for example, and created space for service users to express their needs without the constraints of existing model or expectations. It was an interactive process over time, where one group passed on the Relay baton (service design) to the other after their input, on an iterative basis. This final section explores the implications of the model for theory and practice.

### Implications for theory

We would highlight three implications for theory from this paper.

*Linking value creation to co-design* The project was developed within the Public Service Logic framework that makes explicit that value creation takes place across the four levels of the PSE and that explores the nature of such value within this framework: value-in-use, value-in-context, value-in-production, and value-in-society (Osborne et al., 2022). A unique element of this framework is ‘value-in-production’. Whilst for commercial services, production is only a precursor to value creation for the customer, the dynamic is somewhat different for public services. The engagement of services users and citizens in the processes of public service design/delivery can create value in their lives, irrespective of the outcomes of the service (Osborne, 2021).

In this case, participants reported both increased digital skills and enhanced self-confidence. The project thus provides evidence that service design in a virtual environment can create such value-in-production. Beyond that the project also validated the application of the PSE approach to value creation. Rather than seeking to design public services as discrete outputs in their own right, it sought to explore needs within the prevailing local ecosystem. It explored how the macro-level (Public Value) aspirations of the project became embedded at the meta (service) and micro (individual) levels, mediated by sub-micro personal/professional beliefs. The asynchronous involvement of the local participants and the service-level staff also helped to make extant the differing value configurations at each of these levels and allowed for their discussion and debate, freed from the pre-existing power dynamics of public service delivery. A PSE approach thus shifts the focus of co-design away from ‘service offerings’ and to the nested needs and experiences of service users within their neighbourhood ecosystem.

*Co-design in a virtual space* Dietrich et al (2018) and Trischler et al (2019) have evolved a seven stage model of F2F co-design with citizens and public service users. These stages are resourcing, planning, recruiting, sensitising, facilitating, reflecting, and building for change. Our study confirmed these stages as appropriate in a virtual environment also—though the virtual context did impose its own logic. The sensitisation and facilitation stages, for example, required not only sensitisation of the participants to co-design processes and their facilitation, but also to the virtual context and to the use of digital tools. This was as challenging for the design team, as for the users involved. This echoes the findings of Kennedy et al (2021) in the field of mental health.

*The Relay model* Knapp (2016) evolved the idea of the ‘design sprint’. This involves a short-term and focussed design process during which design-driven innovation processes are conducted. In contrast, our work has evolved a Relay model of co-design. This is where both public services users and professional and care staff are involved in the co-design process, but asynchronously, with the work conducted by one group passed onto the other group in iterative fashion. This model has been discussed in detail above.

In one sense, this was a pragmatic solution to the constraints imposed by the pandemic—F2F co-design was simply not possible. However, as the model evolved it had two significant strengths in its own right. First, it freed service users from the dominance of care staff (however unintentional or well meaning). With appropriate mentoring and facilitation from the design team, it mediated the impact of the pre-existing power imbalances between service users and public service staff. Second, by separating the stages of co-design, it freed users to express their needs in an open and uninhibited way, rather than being filtered through resource constraints at an early and premature stage. This liberated the co-design process to explore needs and translate them into offerings prior to resource discussions.

This is not to say that the model did not have limitations. It was time-consuming because of the iterative stages involved, potentially expensive because of the staffing requirements, and it did minimise the potential for creative interactions between service users and care staff. Nonetheless, these limitations were balanced by the creativity released by the relay model. As Nambisan et al. (2018) have noted in the field of digital innovation management, such virtual co-design can lead to a reframing of the challenges of service delivery and to genuinely new sense-making.

### Implications for practice

Six issues have emerged from this analysis.

*Co-design and user empowerment* A key lesson here was that co-design is not a process of user empowerment. They are undoubtedly overlapping processes—but both can occur without the other. Rather it is one of balancing and synthesising user needs/expectations together with the professional expertise of care staff and the inevitable resource constraints of public service delivery. As Dietrich et al (2018) have noted, the process can be empowering for all parties, but does not (necessarily) privilege one above another.

*The pace of digital transformation and the pandemic* Digital transformation and the migration to a virtual sphere is a defining factor of data-driven innovation in public services (Pencheva et al., 2020). By pivoting co-design from a F2F to a virtual setting, this project mirrored the challenges for the services it was seeking to create/improve. The digital interactions within the co-design process become a testbed for the opportunities and challenges of digital services. This required the facilitators to capture the lessons of these interactions and to ensure that they were integrated into the evolving public service offerings.

Further, the pandemic has accelerated this process, making digital and virtual interactions far more acceptable than previously might have been the case (Kennedy et al.,; 2021). It remains to be seen this level of acceptability is maintained as the pandemic recedes, but it has given a profound surge forward for virtual co-design. There are, of course, dangers in extreme ‘digi-enthusiasm’—in assuming that digital solutions are the default choice in all circumstances. Nonetheless, the pandemic has freed thinking about the role of the virtual in co-design in a way that would have been unthinkable previously. It remains to be seen whether ‘co-motion’, the concept of ‘moving forward together’ (Westoby & Dowling, 2013) is ultimately constrained or enabled by a virtual environment for co-design.

*Operational management of co-design in a virtual space* This project revealed three operational processes that are essential to facilitate effective co-design in a virtual space. First, and most fundamentally, the virtual does not replace the real. Rather it supports it. Human facilitation is essential to humanise and to make co-design work in a virtual environment. Certainly, in this project, the use of facilitators for the co-design sessions was essential to their effectiveness in the virtual space. The sensitisation issue identified by Trischler et al (2019) in their co-design process for public services is also important. However, whilst they talk about sensitisation in the most general terms, of sensitising public service users to co-design processes, the context here is about sensitising users to the virtual environment and the digital tools that they will use. Most/all of these tools will be entirely unfamiliar to them, thus time is required for this sensitisation.

Second, it is also imperative to use both a robust virtual environment that is not prone to collapse, and digital tools that are intuitive for participants. In this case, ‘Mural’ was a highly intuitive tool that users could understand and engage with, though there are others. Strong password protection for the virtual environment also strengthened the preparedness of participants to engage in open discussion.

Finally, an element of ‘Zoom fatigue’ became apparent for the service user and care staff participants in the co-design process. Lengthy engagement in virtual and digital environments is tiring and can be alienating. It is vital to ensure regular respite for participants from virtual engagement.

*The choice of lead users* The choice of the lead users to involve in this process is a key issue. It requires a strong knowledge of the local community of public service users. Inevitably, these individuals will want to become involved from a mixture of altruism and self-interest and these motivations need to be balanced if the process is to be effective.

*Balancing needs and resources* On the one hand, it is essential to keep the early stages of the co-design process as open as possible, to explore user needs openly and freed from prior service expectations and configurations. On the other hand, resource constraints have to be inevitably acknowledged in turning expressed needs into service offerings. The art of virtual co-design is in not allowing the one imperative to crowd out the other.

*Virtual co-design with elderly people* This has its own dynamics. We believe that the *Relay* model addressed the four challenges of co-design with elderly people discussed earlier. It emphasises the need for prior sensitisation of participants to the virtual context, the need to create virtual processes that are congruent to the cognitive skills of participants, the need to focus on process and personal experiences rather than the artefacts of individual services, and the need to address the power imbalances between elderly people and care staff. However, it is not a ‘pro forma’ process that can be applied in an undifferentiated way to all circumstances. It always needs to be situated with the local neighbourhood ecosystems of participants. This is a key lesson of this project.

## Final reflections

This paper has reported the transformation of a co-design project from a F2F to a virtual format. It is not suggested here that either face-to-face or virtual co-design is superior to the other. They both have different contingencies, opportunities, and challenges. What this evaluation has highlighted is the distinctive opportunities and challenges that virtual co-design can present. It is important to emphasise though that this is an exploratory case study of one virtual co-design process. Further research is required to validate the findings of this research and to deepen our understanding of the contingencies of virtual co-design.

The transformation examined here required the co-design team to confront directly many of the implicit challenges of public service co-design. These included the impact of location and disability upon co-design, the import of differential power relationships and of variable skills and information levels between service users and care and professional staff, and, perhaps most importantly—the situating of co-design within an ecosystem perspective, where value creation is a result not of simple dyadic relationships but of nested and interacting relationships. In an increasingly virtual and digital world, it also made the challenge of digital engagement and service delivery a part of the co-design process itself. The resultant *Relay* model was certainly not perfect. However, it did provide a framework to address these challenges and to move forward the theory and practice of co-design in a virtual environment.

## Footnotes

1 https://www.ukri.org/our-work/our-main-funds/industrial-strategy-challenge-fund/ageing-society/healthy-ageing-challenge/.

2 http://mural.co.uk.

3 It was possible only to interview a sample of Blackwood staff as some had moved onto other employment and were no longer contactable.
